# AB Blood System Phenotypes Are Not Associated with *Leishmania infantum* Infection or Seropositivity in Cats from Italy

**DOI:** 10.3390/pathogens15060643

**Published:** 2026-06-17

**Authors:** Eva Spada, Federica Bruno, Germano Castelli, Roberta Perego, Noemi Cerutti, Fabrizio Vitale, Vito Biondi, Luciana Baggiani, Daniela Proverbio

**Affiliations:** 1 Veterinary Transfusion Research Laboratory, Department of Veterinary Medicine and Animal Sciences, University of Milan, 26900 Lodi, Italy; nmcerruti@gmail.com (N.C.); luciana.baggiani@unimi.it (L.B.); daniela.proverbio@unimi.it (D.P.); 2 Centro di Referenza Nazionale per le Leishmaniosi, Istituto Zooprofilattico Sperimentale della Sicilia A. Mirri, 90129 Palermo, Italy; federica.bruno@izssicilia.it (F.B.); germano.castelli@izssicilia.it (G.C.); fabrizio.vitale@izssicilia.it (F.V.); 3 Department of Veterinary Sciences, University of Messina, 98168 Messina, Italy; vito.biondi@unime.it

**Keywords:** cat, feline leishmaniosis, *Leishmania infantum*, blood phenotype, AB blood system, IFAT, qPCR, Italy

## Abstract

Feline leishmaniosis (FeL) caused by *Leishmania infantum* is increasingly recognized in endemic areas, but factors influencing susceptibility in cats remain incompletely understood. Because blood group antigens may modulate host–pathogen interactions, this study evaluated whether feline AB blood system phenotypes are associated with *L. infantum* seropositivity and/or molecular positivity in cats from Italy. Exploratory analyses further assessed whether blood phenotype was associated with the magnitude of indirect fluorescent antibody test (IFAT) antibody titres or with real-time PCR (qPCR) parasite load. In this retrospective cross-sectional study, cats were classified as *L. infantum*-positive when they had an IFAT titre ≥1:80 and/or a positive qPCR on blood or lymph node aspirates. Feline AB blood typing was performed by tube agglutination, with type B and AB samples confirmed by immunochromatographic testing and back typing. A total of 706 cats were included. Overall, 67/706 cats (9.5%) were classified as *L. infantum*-positive. Blood phenotype distribution was 83.1% type A, 10.1% type B, and 6.8% type AB. *L. infantum* positivity was detected in all three phenotypes, and no evidence of association was found between blood phenotype and *L. infantum* positivity, IFAT seropositivity, qPCR positivity, IFAT titre, or qPCR parasite load. After adjustment for region, blood phenotype remained not significantly associated with *L. infantum* positivity. These findings suggest that feline AB blood system phenotypes were not associated with *L. infantum* infection in this feline cohort. Future studies should investigate whether blood phenotype may influence other aspects of FeL, such as clinical expression or disease outcome.

## 1. Introduction

Feline leishmaniosis (FeL), caused by *Leishmania infantum*, is an emerging vector-borne infection increasingly reported in endemic Mediterranean areas, where it is transmitted by phlebotomine sand flies and represents an important vector-borne pathogen both for animals and humans. In cats, serological positivity indicates exposure and a detectable humoral response, whereas molecular positivity provides evidence of parasite DNA; therefore, combining serological and molecular methods can improve epidemiological assessment while recognizing that these tests measure different aspects of exposure and infection [1,2].

Although dogs are considered the main domestic reservoir host, cats can also be infected, may develop clinical disease, and may contribute to the epidemiology of infection [1,2]. In Italy, FeL has been documented in both endemic southern regions and northern areas, and recent epidemiological studies have confirmed that exposure and infection can be detected across heterogeneous feline populations by combining serological and molecular methods [3,4,5,6].

The feline AB blood system is the main erythrocyte antigen system of clinical relevance in cats. Its phenotypes are unevenly distributed across breeds and geographical areas, and naturally occurring alloantibodies make accurate blood typing essential in feline transfusion medicine [7,8,9]. Beyond their transfusion relevance, blood group antigens are biologically plausible host factors in infectious diseases because cell-surface carbohydrate antigens may influence adhesion, recognition, and immune interactions [10,11]. Associations between blood groups and susceptibility to infection have been widely investigated in human medicine, whereas comparable data in cats are still scarce.

Based on this background, the present research aimed to investigate whether feline AB blood system phenotypes are associated with *L. infantum* seropositivity and/or molecular positivity in cats from Italy. We hypothesized that if blood phenotype influenced susceptibility or host–parasite interactions, the prevalence of seropositivity and/or *L. infantum* DNA detection would differ among cats with phenotypes A, B, or AB.

## 2. Materials and Methods

### 2.1. Study Design and Animals

This retrospective cross-sectional study analyzed cats from Italy for which both feline AB blood phenotype and *L. infantum* infection data were available in the study database generated for a previous epidemiological study on FeL [6]. The new analyses specifically concern the feline AB blood phenotype in relation to *L. infantum* seropositivity and/or molecular positivity; these blood-phenotype association analyses were not reported in the previous publication. Evaluated cats originated from two Italian regions, Lombardy in northern Italy and Sicily in southern Italy. When available, the database also included signalment and epidemiological information: origin (north or south of Italy), lifestyle (client-owned, shelter of stray colony cats), gender (male or female), and breed (domestic shorthair—DSH or longhair—DLH or other breeds).

### 2.2. Blood Typing

Feline AB blood typing was performed using the tube agglutination technique as previously described [12,13]. *Triticum vulgaris* lectin was used as an anti-B reagent, and type B plasma as an anti-A reagent. All samples identified as type B and AB by tube agglutination were confirmed by both an immunochromatographic test (Lab Test A + B typing, Alvedia, Limonest, France) and a back typing (alloantibody) test, as previously described [13].

### 2.3. Indirect Fluorescence Antibody Test (IFAT)

Anti-*Leishmania infantum* IgG antibodies were assessed by IFAT, following the WOAH Terrestrial Manual recommendations for leishmaniosis [14] and in accordance with previously reported procedures [6]. Fixed *Leishmania* promastigotes belonging to the WHO reference strain MHOM/IT/80/IPT1 (Bio-Mérieux, Marcy l’Etoile, France) were used as antigenic substrate. Cat serum samples were subjected to two-fold serial dilutions in PBS, ranging from 1:40 to 1:5120, and then applied to antigen-coated slides. After incubation for 30 min at 37 °C, the slides were washed and processed with a fluorescein-labelled goat anti-cat IgG antibody (Sigma-Aldrich, St. Louis, MO, USA) recognizing the whole molecule and conjugated to fluorescein isothiocyanate (FITC), diluted 1:200 in phosphate-buffered saline (PBS). Positive and negative control sera were included in each analytical session. The slides were evaluated using a Leica DM 4000B fluorescence microscope (Leica Microsystems Srl, Buccinasco, Milan, Italy) Samples showing antibody titers of 1:80 or higher were classified as positive [1,15].

### 2.4. Real-Time PCR (qPCR)

Genomic DNA was purified from ethylenediaminetetraacetic acid (EDTA)-anticoagulated whole blood and/or lymph node aspirate samples using the PureLink Genomic DNA Mini Kit (Thermo Fisher Scientific K182002, Waltham, MA, USA), according to the manufacturer’s protocol. Quantitative real-time PCR was carried out on a QuantStudio 3 system (Life Technologies, Waltham, MA, USA), as previously described [16]. Each reaction was prepared in a final volume of 20 µL, including 10 µL of SsoAdvanced Universal Probes Supermix (Bio-Rad, Hercules, CA, USA), 0.25 µM QLeish probe, 0.3 µM of each primer, and 2 µL of extracted DNA adjusted to 10 ng/µL. For absolute quantification, a standard curve was generated using 10-fold serial dilutions of *L. infantum* DNA, corresponding to concentrations from 1 × 10^6^ to 1 parasite/mL. The amplification protocol consisted of an initial denaturation at 95 °C for 10 min, followed by 40 cycles of denaturation at 95 °C for 15 s and annealing/extension at 60 °C for 35 s.

Not all cats were tested using both whole blood and lymph node aspirate, and qPCR was performed according to sample availability. When both specimens were available, cats were considered qPCR-positive if either specimen yielded a positive result. For parasite-load analyses, when more than one numerical qPCR value was available for the same cat, the highest value was retained.

### 2.5. Definition of L. infantum Seropositivity and/or Molecular Positivity

Because IFAT detects anti-*L. infantum* IgG antibodies and therefore indicates exposure/seropositivity rather than active infection, cats were classified as *L. infantum*-positive when they had an IFAT antibody titre ≥1:80 and/or a positive qPCR result on blood or lymph node aspirates. Therefore, IFAT-positive cats are referred to as seropositive, qPCR-positive cats as molecularly positive, and the combined endpoint as *L. infantum* positivity or evidence of exposure and/or infection.

### 2.6. Statistical Analysis

Descriptive statistics were calculated for the overall study population and according to *L. infantum* status. Categorical variables were summarized as numbers and percentages, whereas age was reported as median and interquartile range (IQR). Associations between categorical variables and *L. infantum* positivity were assessed using Pearson’s chi-square test or Fisher’s exact test, as appropriate. The same approach was used to test the association between blood phenotype and IFAT seropositivity and between blood phenotype and qPCR positivity. Odds ratios (ORs) with 95% confidence intervals (95% CIs) were calculated for selected binary comparisons, including A versus non-A, B versus non-B, and AB versus non-AB phenotypes.

Additional exploratory analyses were performed to evaluate whether the AB blood group system was associated with the magnitude of the anti-*Leishmania* antibody response or with qPCR parasite burden. IFAT positivity was defined a priori as an endpoint titre ≥1:80; negative results and titres of 1:40 were considered IFAT-negative for binary analyses. Endpoint IFAT titres were additionally analyzed as an ordinal variable after retaining the negative and 1:40 categories, and IFAT-positive cats were analyzed separately after log2 transformation of endpoint dilution values.

For parasite-load analyses, qPCR results on blood and lymph node were used. Parasite load was analyzed after log10 transformation. Comparisons between type A and non-A cats were performed using Mann–Whitney U tests, whereas three-group comparisons among A, B, and AB cats were performed using Kruskal–Wallis tests. Because of the low number of qPCR-positive type B and AB cats, parasite-load analyses were considered exploratory. Because both blood phenotypes and FeL have significantly different distributions in North and South Italy [6,13], to assess the potential confounding effect of geographical origin, logistic regression was performed with *L. infantum* positivity as the dependent variable and blood phenotype and region as explanatory variables. The overall contribution of blood phenotype was assessed by likelihood-ratio testing. A *p*-value < 0.05 was considered statistically significant. All statistical analyses were performed using MedCalc^®^ Statistical Software version 23.5.5 (MedCalc Software Ltd., Ostend, Belgium).

## 3. Results

### 3.1. Study Population

A total of 706 cats were included. Data on origin, lifestyle, gender, and breed are summarized in Table 1. Exact age was available for 402 cats, with a median age of 2.0 years (IQR, 1.0–6.0 years).

### 3.2. L. infantum Status

Overall, 67/706 cats were classified as *L. infantum*-positive, yielding an overall positivity of 9.5%. Among the 67 *L. infantum*-positive cats, 50/67 (74.6%) were positive by IFAT only, 8/67 (11.9%) were positive by qPCR only, and 9/67 (13.4%) were positive by both IFAT and qPCR.

IFAT serostatus was available for 682 cats. Most cats were seronegative (<1:40; 562/687, 82.4%) or had low titres below the positivity cut-off (1:40; 61/687, 8.9%). Among IFAT-seropositive cats (≥1:80; 59/687, 8.6%), titres were 1:80 in 37 cats (5.4%), 1:160 in 16 cats (2.3%), 1:320 in three cats (0.4%), 1:1280 in one cat (0.1%), and 1:2560 in two cats (0.3%); no cat had a titre of 1:640.

qPCR status, based on blood and/or lymph-node analysis, was available for 679 cats. Specifically, blood qPCR data were available for 674 cats and lymph-node qPCR data for 276 cats. qPCR positivity, based on blood and/or lymph-node results, was detected in 17/679 tested cats (2.5%). Overall, the median parasite load was 15 parasites/mL (IQR, 10–80). Quantifiable blood qPCR values were available for 13 cats, with a median of 20 parasites/mL (IQR, 10–250), whereas quantifiable lymph-node aspirate qPCR values were available for four cats, with a median of 11.5 parasites/mL (IQR, 7.5–20).

### 3.3. Blood Phenotype Distribution

The distribution of AB blood phenotypes in the analyzed population was as follows: type A, 587/706 (83.1%); type B, 71/706 (10.1%); and type AB, 48/706 (6.8%). The distribution of blood phenotypes differed significantly between regions (χ^2^ = 50.05; *p* < 0.001): in Lombardy, 369/402 cats were phenotype A, 21/402 were phenotype B, and 12/402 were phenotype AB; in Sicily, 218/304 cats were phenotype A, 50/304 were phenotype B, and 36/304 were phenotype AB.

### 3.4. Association Between AB Blood Phenotypes and L. infantum Seropositivity and/or Molecular Positivity

**Figure 1:**
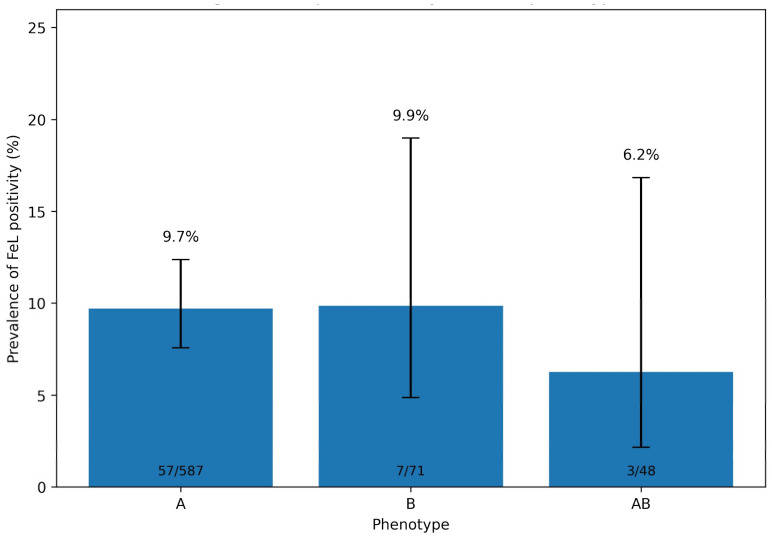
Prevalence of feline *Leishmania infantum* positivity according to AB blood phenotypes. Bars represent prevalence percentages, and error bars indicate 95% confidence intervals.

*L. infantum* positivity was detected in cats of all three blood phenotypes. Specifically, positivity was observed in 57/587 phenotype A cats (9.7%), 7/71 phenotype B cats (9.9%), and 3/48 phenotype AB cats (6.2%). No significant association was found between blood phenotypes and *L. infantum* positivity (*p* = 0.729, Figure 1).

After adjustment for region, blood phenotype remained not significantly associated with *L. infantum* positivity (overall effect of blood phenotype: likelihood-ratio χ^2^ = 2.22; *p* = 0.329). In the adjusted model, type B cats did not differ significantly from type A cats (adjusted OR = 0.77; 95% CI, 0.33–1.79; *p* = 0.538), and type AB cats also did not differ significantly from type A cats (adjusted OR = 0.45; 95% CI, 0.13–1.52; *p* = 0.198).

Serological positivity did not differ significantly among blood phenotypes (*p* = 0.774). qPCR positivity on blood and/or lymph-node detected in 17/679 was likewise not associated with blood phenotype (*p* = 0.423) (Table 2).

### 3.5. IFAT Titre Distribution and qPCR Parasite Load According to AB Blood Phenotype

IFAT endpoint titre distribution did not differ significantly according to blood phenotype. The distribution of negative, 1:40, 1:80, and ≥1:160 titres was 467, 48, 30, and 19 in type A cats; 53, 10, 5, and 2 in type B cats; and 42, 3, 2, and 1 in type AB cats, respectively (Table 3). Comparison of IFAT titres showed no significant difference between type A and non-A cats (Mann–Whitney U *p* = 0.607), nor among A, B, and AB cats (Kruskal–Wallis *p* = 0.248). When the analysis was restricted to IFAT-positive cats (titre ≥ 1:80), the median titre was 1:80 in both phenotype A and non-A cats (range, 1:80–1:2560 and 1:80–1:320, respectively; Mann–Whitney U *p* = 0.629).

Quantifiable qPCR parasite-load available for 17 cats with positive blood and/or lymph-node qPCR included 16 type A cats and one type B cat; no type AB cat had a quantifiable qPCR parasite load (Table 4). Median parasite load among type A cats was 12.5 (range, 5–3000; median log10 load, 1.09). The only non-A cat with a quantifiable parasite load was phenotype B, which had a load of 84,400 (log10 load, 4.93). This isolated value was not interpreted as evidence of a phenotype-related effect because only one non-A cat had quantifiable parasite load; the comparison was not statistically significant and was not robust (Mann–Whitney U *p* = 0.123).

## 4. Discussion

In this cohort of cats from Italy, no evidence of association was detected between AB blood system phenotype and *L. infantum* positivity, IFAT seropositivity, qPCR positivity, IFAT endpoint titre distribution, or qPCR parasite load (Table 2, Table 3 and Table 4). *L. infantum* positivity was detected in cats of all three phenotypes, with the proportions of positive animals similar across A, B, and AB cats, and the estimated ORs did not indicate increased risk for any phenotypes. After adjustment for region, blood phenotype remained not significantly associated with *L. infantum* positivity. These findings support the interpretation that feline AB system erythrocyte antigens and related naturally occurring alloantibodies were not associated with *L. infantum* seropositivity and/or molecular positivity in the investigated population.

The biological rationale for exploring this association was plausible [17]. Blood group antigens are surface carbohydrate structures that may modulate host–pathogen interactions and, in humans, have long been investigated as potential determinants of susceptibility to infectious diseases [10,11]. In cats, however, the AB blood system has been studied mainly in relation to transfusion compatibility, neonatal isoerythrolysis, and alloantibody-mediated incompatibility rather than infectious disease risk [7,9]. The role of feline AB blood group phenotypes and genotypes in modulating host–pathogen relationships remains poorly characterized, as only a limited number of studies have addressed this topic in recent years. To date, no association has been demonstrated between AB blood types and infections caused by *Toxoplasma gondii* [18], feline retroviruses, including feline immunodeficiency virus (FIV) and feline leukemia virus (FeLV) [19], or feline coronavirus [20]. Conversely, significant relationships have been reported for the AB phenotype in cats infected with severe acute respiratory syndrome coronavirus 2 (SARS-CoV-2) [21] and for the *Ab* genotype in association with *Mycoplasma haemofelis* infection [22]. Nevertheless, the biological mechanisms that may explain these findings have not yet been clarified.

The absence of a detectable association in the present study suggests that, if the feline AB blood group system has any influence on *L. infantum* infection, its effect is likely to be small compared with other host, vector, and environmental aspects [2]. This result is similar to those in studies published for human visceral leishmaniosis (VL). No significant differences were found between patients with American VL and controls, indicating that ABO blood group type is not an important factor in the development of VL [23]. Similarly, the ABO-Rh blood groups were not associated with the occurrence of VL in Iranian patients [24].

The lack of association between blood phenotype and FeL status is also consistent with the broader complexity of feline *L. infantum* epidemiology. Previous studies have shown that FeL prevalence varies according to geographical area, cat population, diagnostic approach, and the biological sample tested [3,5,25,26,27].

The regional distribution of blood phenotypes was uneven, with phenotype B and AB cats proportionally more frequent in Sicily than in Lombardy [13]. For this reason, the region was considered a potential confounder and was included in the adjusted logistic model. The adjusted analysis confirmed that blood phenotype was not significantly associated with *L. infantum* positivity.

The additional analyses of IFAT endpoint titres provided no evidence that AB blood phenotype influenced the magnitude of the anti-*Leishmania* humoral response (Table 3). IFAT positivity was defined as a titre ≥1:80, while titres of 1:40 were retained as a separate low-titre category for ordinal analyses. Neither the overall ordinal distribution of titres nor the distribution among IFAT-positive cats differed among phenotypes. Therefore, in this evaluation, blood phenotype was not associated with a stronger or weaker measurable antibody response to *L. infantum*.

Similarly, qPCR parasite-load analysis did not support a clear effect of AB blood phenotype on parasite burden. However, this analysis should be considered descriptive and exploratory only (Table 4). Quantifiable parasite load, in fact, was available in 17 cats, almost all of which were type A; only one non-A cat, belonging to phenotype B, had a quantifiable qPCR load, and no type AB cat had quantifiable parasite DNA. Although the single type B cat showed a high parasite-load value, this isolated observation cannot be interpreted as evidence of a group-level effect. Larger datasets, including more qPCR-positive type B and type AB cats, would be required to determine whether blood phenotype influences parasite burden among positive cats.

From a diagnostic perspective, the combined use of IFAT and qPCR likely strengthened FeL case identification. IFAT remains one of the most used serological methods in FeL and is useful in both clinical and epidemiological settings, although serological and molecular tests detect partially different aspects of infection and do not always overlap [1,28]. In the present study, most cats were identified by IFAT only, whereas a smaller proportion were detected exclusively by qPCR or by both methods. This pattern is in line with published guidelines on FeL reporting that the combination of serological and molecular tests improves detection of exposure and infection in field investigations [1,2].

This study has some limitations. First, the relatively small number of type B and AB cats, however, reflects their natural distribution in the general feline population [8] and especially the small number of FeL-positive or qPCR-positive cats with non-A phenotypes, which reduced the precision of risk estimates and the power of parasite-load comparisons. Therefore, the findings should be interpreted as the absence of evidence of an association rather than definitive evidence of no effect. Second, because of the retrospective nature of the database, some variables were incompletely recorded, and not all cats underwent the same full diagnostic work-up. In particular, the low number of qPCR-positive cats prevented a robust assessment of the association between blood phenotypes and parasite burden. In addition, qPCR testing was performed in only a subset of cats and on different sample types. This is relevant because diagnostic sensitivity may differ between blood and lymph node samples, with lymph node testing potentially detecting tissue-associated infection more reliably than blood, especially in cases with low circulating parasite loads [1,29]. Third, the study was not designed to assess whether blood phenotype influences clinical severity, longitudinal disease evolution, or detailed clinicopathological abnormalities among infected cats.

Despite these limitations, the study has notable strengths, including a relatively large feline sample size for this field, the inclusion of cats from two Italian regions with different epidemiological contexts, confirmatory blood typing procedures for phenotype B and AB samples, and the use of both serological and molecular criteria to define *L. infantum* status. Altogether, the data support the practical conclusion that the feline phenotype of AB blood system was not associated with *L. infantum* positivity, serological response intensity, or qPCR parasite burden in this cohort, while acknowledging that effects cannot be excluded on clinical severity, disease evolution, or clinicopathological abnormalities among infected cats.

## 5. Conclusions

In conclusion, no evidence of association was detected between feline AB blood system phenotypes and *L. infantum* positivity, IFAT serostatus, IFAT titre magnitude, qPCR positivity, or qPCR parasite load in this cohort of cats from Italy. Future studies should investigate whether blood phenotype may influence other aspects of FeL, such as clinical expression or disease outcome.

## Figures and Tables

**Table 1:** Main characteristics of a feline population of 706 cats from Italy, evaluated for an association between blood phenotypes and *Leishmania infantum* status.

Variable	Category	n (%)
Origin n = 706	Lombardy	402 (56.9%)
	Sicily	304 (43.1%)
Lifestyle n = 549	Client-owned	153 (27.9%)
	Stray-colony	335 (61.0%)
	Shelter	61 (11.1%)
Gender n = 556	Male	282 (50.7%)
	Female	274 (49.3%)
DSH/DLH n = 441	Yes	409 (92.7%)
	No	32 (7.3%)
Blood phenotype n = 706	A	587 (83.1%)
	B	71 (10.1%)
	AB	48 (6.8%)

DSH/DLH: domestic shorthair-longhair.

**Table 2:** Association between *Leishmania infantum* positivity, IFAT seropositivity, qPCR positivity, and feline AB blood phenotypes.

Analysis	Blood Phenotype	Positive n (%)	Negative n (%)	*p*-Value
*L. infantum* positivity n = 706	A	57/587 (9.7%)	530/587 (90.3%)	0.729
	B	7/71 (9.9%)	64/71 (90.1%)	
	AB	3/48 (6.2%)	45/48 (93.8%)	
IFAT status n = 682	A	49/564 (8.7%)	515/564 (91.3%)	0.774
	B	7/70 (10.0%)	63/70 (90.0%)	
	AB	3/48 (6.2%)	45/48 (93.8%)	
qPCR status n = 679	A	16/565 (2.8%)	549/565 (97.2%)	0.423
	B	1/69 (1.4%)	68/69 (98.6%)	
	AB	0/45 (0.0%)	45/45 (100.0%)	

IFAT: indirect fluorescent antibody test; qPCR: real-time polymerase chain reaction.

**Table 3:** *Leishmania infantum* IFAT endpoint titre distribution according to feline AB blood phenotype.

Blood Phenotype	<1:40	1:40	1:80	≥1:160	IFAT ≥ 1:80, n (%)
A	467	48	30	19	49/564 (8.7%)
B	53	10	5	2	7/70 (10.0%)
AB	42	3	2	1	3/48 (6.2%)
non-A (B + AB)	95	13	7	3	10/118 (8.5%)

**Table 4:** Exploratory descriptive analyses evaluating whether the AB blood group system phenotype was associated with qPCR parasite burden for *Leishmania infantum* in cats in Italy.

Blood Phenotype	n with Quantifiable qPCR Load	Median Load	Range	Median log10 Load
A	16	12.5	5–3000	1.09
B	1	84,400	84,400–84,400	4.93
AB	0	-	-	-
non-A (B + AB)	1	84,400	84,400–84,400	4.93

## Data Availability

The data presented in this study are available from the corresponding authors upon reasonable request, subject to institutional and privacy restrictions.

## Abbreviations

The following abbreviations are used in this manuscript:

FeL	Feline leishmaniosis
IFAT	Indirect fluorescent antibody test
qPCR	Real-time polymerase chain reaction
SARS-CoV-2	Severe acute respiratory syndrome coronavirus 2
VL	Visceral leishmaniosis
FIV	Feline immunodeficiency virus
FeLV	Feline leukemia virus
